# Individualized Constellation of Killer Cell Immunoglobulin-Like Receptors and Cognate HLA Class I Ligands that Controls Natural Killer Cell Antiviral Immunity Predisposes COVID-19

**DOI:** 10.3389/fgene.2022.845474

**Published:** 2022-02-22

**Authors:** Stalinraja Maruthamuthu, Karan Rajalingam, Navchetan Kaur, Maelig G. Morvan, Jair Soto, Nancy Lee, Denice Kong, Zicheng Hu, Kevin Reyes, Dianna Ng, Atul J. Butte, Charles Chiu, Raja Rajalingam

**Affiliations:** ^1^ Immunogenetics and Transplantation Laboratory, Department of Surgery, University of California, San Francisco, San Francisco, CA, United States; ^2^ Charles E. Schmidt College of Medicine, Florida Atlantic University, Boca Raton, FL, United States; ^3^ Bakar Computational Health Sciences Institute, University of California, San Francisco, San Francisco, CA, United States; ^4^ Department of Pediatrics, University of California, San Francisco, San Francisco, CA, United States; ^5^ UCSF-Abbott Viral Diagnostics and Discovery Center, Department of Laboratory Medicine, University of California, San Francisco, San Francisco, CA, United States; ^6^ Department of Medicine, University of California, San Francisco, San Francisco, CA, United States; ^7^ Department of Pathology, University of California, San Francisco, San Francisco, CA, United States; ^8^ Helen Diller Family Comprehensive Cancer Center, University of California, San Francisco, San Francisco, CA, United States; ^9^ Department of Laboratory Medicine, University of California, San Francisco, San Francisco, CA, United States

**Keywords:** COVID-19 susceptibility, NK cells, KIR-HLA association, KIR receptors, HLA association, SARS-CoV-2 susceptibility, antiviral immunity, COVID-19 host genetics

## Abstract

**Background:** The severe acute respiratory syndrome coronavirus-2 (SARS-CoV-2) infection causes coronavirus disease-2019 (COVID-19) in some individuals, while the majority remain asymptomatic. Natural killer (NK) cells play an essential role in antiviral defense. NK cell maturation and function are regulated mainly by highly polymorphic killer cell immunoglobulin-like receptors (KIR) and cognate HLA class I ligands. Herein, we tested our hypothesis that the individualized KIR and HLA class I ligand combinations that control NK cell function determine the outcome of SARS-CoV-2 infection.

**Methods:** We characterized KIR and HLA genes in 200 patients hospitalized for COVID-19 and 195 healthy general population controls.

**Results:** The KIR3DL1^+^HLA-Bw4^+^ [Odds ratio (OR) = 0.65, *p* = 0.03] and KIR3DL2^+^HLA-A3/11^+^ (OR = 0.6, *p* = 0.02) combinations were encountered at significantly lower frequency in COVID-19 patients than in the controls. Notably, 40% of the patients lacked both of these KIR^+^HLA^+^ combinations compared to 24.6% of the controls (OR = 2.04, *p* = 0.001). Additionally, activating receptors KIR2DS1^+^KIR2DS5^+^ are more frequent in patients with severe COVID-19 than patients with mild disease (OR = 1.8, *p* = 0.05). Individuals carrying KIR2DS1^+^KIR2DS5^+^ genes but missing either KIR3DL1^+^HLA-Bw4^+^ combination (OR = 1.73, *p* = 0.04) or KIR3DL2^+^HLA-A3/11^+^ combination (OR = 1.75, *p* = 0.02) or both KIR3DL1^+^HLA-Bw4^+^ and KIR2DL2^+^HLA-A3/11^+^ combinations (OR = 1.63, *p* = 0.03) were more frequent in the COVID-19 cohort compared to controls.

**Conclusions:** The absence of KIR3DL1^+^HLA-Bw4^+^ and KIR3DL2^+^HLA-A3/11^+^ combinations presumably yields inadequate NK cell maturation and reduces anti-SARS-CoV-2 defense, causing COVID-19. An increased frequency of KIR2DS1^+^KIR2DS5^+^ in severe COVID-19 patients suggests vigorous NK cell response triggered via these activating receptors and subsequent production of exuberant inflammatory cytokines responsible for severe COVID-19. Our results demonstrate that specific KIR-HLA combinations that control NK cell maturation and function are underlying immunogenetic variables that determine the dual role of NK cells in mediating beneficial antiviral and detrimental pathologic action. These findings offer a framework for developing potential host genetic biomarkers to distinguish individuals prone to COVID-19.

## Introduction

Since the emergence of severe acute respiratory syndrome coronavirus (SARS-CoV) in 2002, the world has experienced the outbreak of middle east respiratory syndrome coronavirus (MERS-CoV) in 2012, and presently of SARS-CoV-2 started in 2019. These repeated episodes make coronaviruses a continuous threat to humanity. Approximately 80% of those infected with SARS-CoV-2 experience mild symptoms such as cough and fever, and do not require hospitalization (https://covid19.who.int). Nearly 50% of hospitalized individuals develop coronavirus disease 2019 (COVID-19) (Drake et al., 2021). The outcomes of SARS-CoV-2 infection cover a broad spectrum ranging from the control of infection to the development of acute respiratory distress syndrome (ARDS), a life-threatening lung injury that leaves breathing difficult, and death, indicating a role for intrinsic host factors in the pathogenesis of COVID-19. Older age, male gender, pre-existing conditions (e.g., hypertension, diabetes, coronary heart disease), ethnic and racial minority (African Americans, Hispanics) render a person more vulnerable to the severe health consequences of SARS-CoV-2 infection (Bhala et al., 2020). Genome-wide association analyses in a large case-control study identified the 3p21.31 gene cluster and ABO blood-group system associated with patients suffering from COVID-19 (Severe Covid-19 GWAS Group et al., 2020). However, the influence of germline-encoded genetic variations in the host that control the immune response to SARS-CoV-2 infection and differential disease manifestations remains unclear.

Natural killer (NK) cells are fast-acting innate lymphocytes that play a central role in early controlling viral infections (Biron et al., 1989; Bjorkstrom et al., 2021), including in SARS-CoV-2 infection (Maucourant et al., 2020). Fruit bats, a natural reservoir of numerous zoonotic viruses including SARS and MERS, remain asymptomatic due to immune genes that largely control NK cell antiviral defense (Pavlovich et al., 2018). The lower NK cell counts were associated with COVID-19 progression and severe symptoms (Osman et al., 2020; Wang et al., 2020), longer duration of viral shedding (Bao et al., 2021), severe COVID-19 illness (Song et al., 2020; Zheng et al., 2020; Bao et al., 2021), and poor patient survival (Bao et al., 2021). The NK cells are enriched in the human lung compared with peripheral blood during COVID-19 (Zhou et al., 2020). Although a reduction in total NK cell counts, the function of NK cells seems to be intensified in COVID-19 patients. More mature (CD56^dim^CD16^+^ phenotype) and memory (CD57^+^CD85j^+^ phenotype) NK cells with increased expression of KIR receptors were found in hospitalized patients with early (Bozzano et al., 2021) and severe COVID-19 illness (Maucourant et al., 2020). These findings collectively indicate a dual role for NK cells in responding to acute SARS-CoV-2 infections but might also contribute to COVID-19 pathology. However, the molecular mechanism underlying such a dual role of NK cells against SARS-CoV-2 infection remains elusive.

**FIGURE 1:**
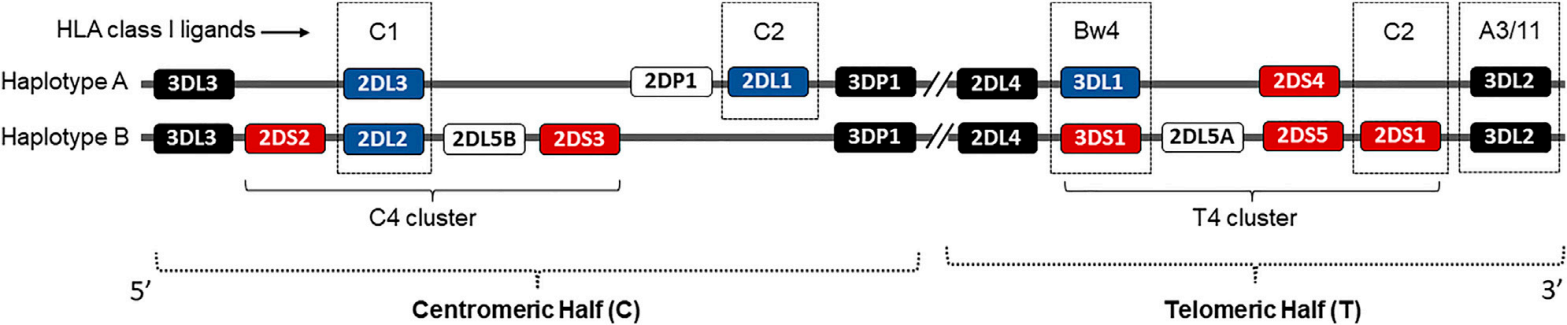
Schematic representation of the KIR genetic complex. Two common KIR haplotypes, A and B, carry contrasting numbers and types of KIR genes. Genes encoding KIR receptors for cognate HLA class I ligands are identified in dotted boxes. Genes encoding activating KIRs are shown in red boxes. The framework genes are shown in dark boxes. The centromeric and telomeric KIR halves and C4 and T4 gene clusters are marked.

Human NK cells use a complex germline-encoded receptor-ligand system that calibrates signals triggered by an array of inhibitory and activating receptors to ensure self-tolerance against healthy cells while killing virally infected target cells (Lanier, 1998; Rajalingam, 2018). NK cells also produce high levels of interferon-γ (IFN-γ) and a wide range of pro-inflammatory cytokines, as well as chemokines (Stetson et al., 2003). The HLA class I binding killer cell immunoglobulin-like receptors (KIR) are the key regulators of human NK cell effector function (Lanier, 1998). Fourteen distinct KIR receptors are defined that trigger either inhibition (3DL1-3, 2DL1-3, 2DL5) or activation (3DS1, 2DS1-5), or both function (2DL4) (Parham, 2005) (Figure 1). The HLA ligands are well defined for inhibitory KIRs, while the ligands for activating KIRs remain elusive. The inhibitory KIR2DL2 and KIR2DL3 receptors bind to group 1 HLA-C (HLA-C1) allotypes, which has an asparagine at amino acid position 80, while KIR2DL1 binds to group 2 HLA-C (HLA-C2) allotypes, which has a lysine at the same position (Winter et al., 1998). The inhibitory KIR3DL1 binds with HLA-Bw4, and KIR3DL2 binds with HLA-A3/11 (Gumperz et al., 1995; Hansasuta et al., 2004). The NK cells expressing KIR3DL1 are more potently inhibited by the HLA-Bw4 subset possessing isoleucine at amino acid position 80 (I80) than those with threonine (T80) (Gumperz et al., 1995). Some activating KIR receptors exhibit high amino acid sequence homology with the corresponding inhibitory KIRs in their extracellular immunoglobulin-like domains. Therefore, these activating KIRs presumably share the HLA class I ligands with inhibitory KIR counterparts. For example, KIR2DS1 and 2DL1 differ by only seven amino acids in their extracellular immunoglobulin-like domains, and therefore, KIR2DS1 is known to bind weakly to HLA-C2 (Chewning et al., 2007; Foley et al., 2008; Hayley et al., 2011; Sivori et al., 2011; Carlomagno et al., 2017). The KIR3DS1 shares the highest sequence homologies with KIR3DL1 in their extracellular immunoglobulin-like domains and therefore is considered binding to HLA-Bw4 (O'Connor et al., 2015; Carlomagno et al., 2017). The KIR2DS2, whose extracellular domain differs from KIR2DL2 and 2DL3 by only three and four amino acids, respectively, binds to HLA-A*11:01 complexed with a vaccinia viral peptide (Liu et al., 2014). Using KIR-Fc fusion protein on HLA class I molecule-conjugated Luminex microbeads, specific alleles of the activating KIR2DS5 were shown to bind HLA-C2 (Blokhuis et al., 2017). The activating KIR2DS3 does not bind any HLA (Saulquin et al., 2003; Hilton et al., 2012).

By interacting with specific cognate HLA class I ligands, the inhibitory KIRs beget NK cell maturation and acquisition of full effector function, developmental programming called “licensing” (Anfossi et al., 2006; Kim et al., 2008). In the absence of inhibitory KIR-HLA interactions, NK cells become hyporesponsive or anergic.

The KIR and HLA gene families are highly polymorphic, and they map to distinct human chromosomes 19 and 6, respectively. The independent segregation of these gene families yields individuals expressing KIR receptors for which they lack HLA class I ligands and vice versa, creating a heterogeneity between individuals in the number and type of KIR-HLA gene composition inherited (Du et al., 2007). Consequently, certain combinations of KIR-HLA variants correlate with susceptibility to diseases as diverse as autoimmunity, viral infections, and cancer (Rajagopalan and Long, 2005; Khakoo and Carrington, 2006; Kucuksezer et al., 2021). We hypothesize that the individualized KIR and cognate HLA class I ligand combinations that control NK cell function determine the outcome of SARS-CoV-2 infection.

## Materials and Methods

### Study Cohorts

The study includes 200 individuals hospitalized for COVID-19 care at the University of California San Francisco Medical Center from March to October 2020. Patients were sub-grouped into mild (*N* = 93) or severe (*N* = 107) according to the Centers for Disease Control and Prevention guidance. Clinical characteristics of COVID-19 patients and clinical groups are provided in Table 1. None of the patients with mild disease needed ICU care or had ARDS or acute organ failure. The study was reviewed and approved by the UCSF Institutional Review Board of human research protection (IRB Number: 20-31107). The control group includes 195 healthy volunteers from the same geographical region collected at the pre-pandemic period.

**TABLE 1:** Demographic and clinical characteristics of the hospitalized COVID-19 patients.

Characteristic	All patients *n* = 200	Mild *n* = 93	Severe *n* = 107	*p*-value	Severe vs mild OR (95% CI)
	% (N)	% (N)	% (N)		
Male gender	56.0 (112)	45.2 (42)	65.4 (70)	0.004	2.3 (0.26–0.77)
Median age (IQR)	55 (45.5–67.5)	52 (43–66)	59 (47–70)		
Race					
White	31.5 (63)	32.3 (30)	30.8 (33)		
African American	10.0 (20)	11.8 (11)	8.4 (9)		
Asian	12.5 (25)	10.8 (10)	14.0 (15)		
Unknown	46.0 (92)	45.2 (42)	46.7 (50)		
Clinical characteristics					
Fever	33.5 (67)	21.5 (20)	43.9 (47)	0.001	2.9 (0.18–0.65)
Hypoxia	67.0 (134)	49.5 (46)	82.2 (88)	0.0001	4.7 (0.11–0.40)
Chest pain	26.0 (52)	29.0 (27)	23.4 (25)		
Caugh	18.0 (36)	20.4 (19)	15.9 (17)		
Pneumonia	67.5 (135)	52.7 (49)	80.4 (86)	0.0001	3.7 (0.15–0.51)
Diarrhea	9.5 (19)	6.5 (6)	12.1 (13)		
Shortness of breath	40.5 (81)	37.6 (35)	43.0 (46)		
Respiratory illness	59.5 (119)	40.9 (38)	75.7 (81)	0.0001	4.5 (0.12–0.41)
Ventilator support	32.0 (64)	10.8 (10)	50.5 (54)	0.0001	8.46 (0.05–0.25)
ICU	24.0 (48)	0.0 (0)	44.9 (48)	0.0004	152.4 (9.2–2519.2)
Acute organ failure	44.0 (88)	0.0 (0)	82.2 (88)	0.0001	848.7 (50.4–14269.5)
ARDS	18.5 (37)	0.0 (0)	34.6 (37)	0.001	99.5 (6.0–1647.8)
Death	10.0 (20)	0.0 (0)	18.7 (20)	0.008	43.8 (2.6–735.4)

%, Percentage of individuals in each category is defined as the number of individuals having the characteristics (N) divided by the number of individuals studied (n) in the study group; OR, odds ratio; CI, confidence interval; IQR, interquartile range.

### KIR and HLA Genotyping

Genomic DNA was extracted from PBMC using the Qiagen DNA extraction kit (Qiagen, Valencia, CA, United States). KIR genotyping was performed using the Luminex-based oligonucleotide probe hybridization method according to the manufacturer’s instructions (One Lambda, Canoga Park, CA, United States). HLA genotyping of COVID-19 patients was done by long-range PCR-based next-generation sequencing (NGS) reagent on the MiSeq sequencer (Illumina, San Diego, CA, United States) per the manufacturer’s recommendations (Omixon, Inc. Budapest, Hungary). The control samples were HLA typed using a commercially available Sequence-Specific Oligonucleotide hybridization kit (LABType^®^ SSO, One Lambda, Canoga Park, CA). We described the KIR genotyping (Sun et al., 2021) and HLA genotyping protocols by NGS (Kong et al., 2021) and SSO (Sun et al., 2021) elaborately in our previous publications.

### KIR and HLA Data Analysis and Statistical Methods

Based on the presence and absence of KIR genes, the KIR genotypes are defined as AA and Bx genotypes. The presence of only KIR3DL3-2DL3-2DL1-2DP1-3DP1-2DL4-3DL1-2DS4-3DL2 genes that are characteristic of A-haplotype are defined as AA genotype, and the presence of all other KIR gene combinations are defined as Bx genotype. The Bx genotypes were further examined for the presence and absence of centromeric and telomeric KIR clusters (Figure 1). The centromeric cluster consists of KIR2DS2-2DL2-2DS3-2DL5 genes and is termed the C4 linkage group. The telomeric cluster consists of KIR3DS1-2DL5-2DS5-2DS1 genes and is termed the T4 linkage group. The percentage of each KIR and HLA class I ligand between the COVID-19 patient and general population was calculated by direct counting (number of individuals positive for the gene divided by the total number of individuals in the group X 100). The distribution of each KIR genotypes, KIR genes, HLA ligands, and KIR-HLA combinations between the study groups were estimated by Pearson chi-square, and a *p*-value of less than 0.05 was considered significant. The odds ratio (OR) was calculated with a 95% confidence interval (CI).

## Results

### Lack of KIR3DL1^+^HLA-Bw4^+^ and KIR3DL2^+^HLA-A3/11^+^ Gene Combinations are Associated With COVID-19

**FIGURE 2:**
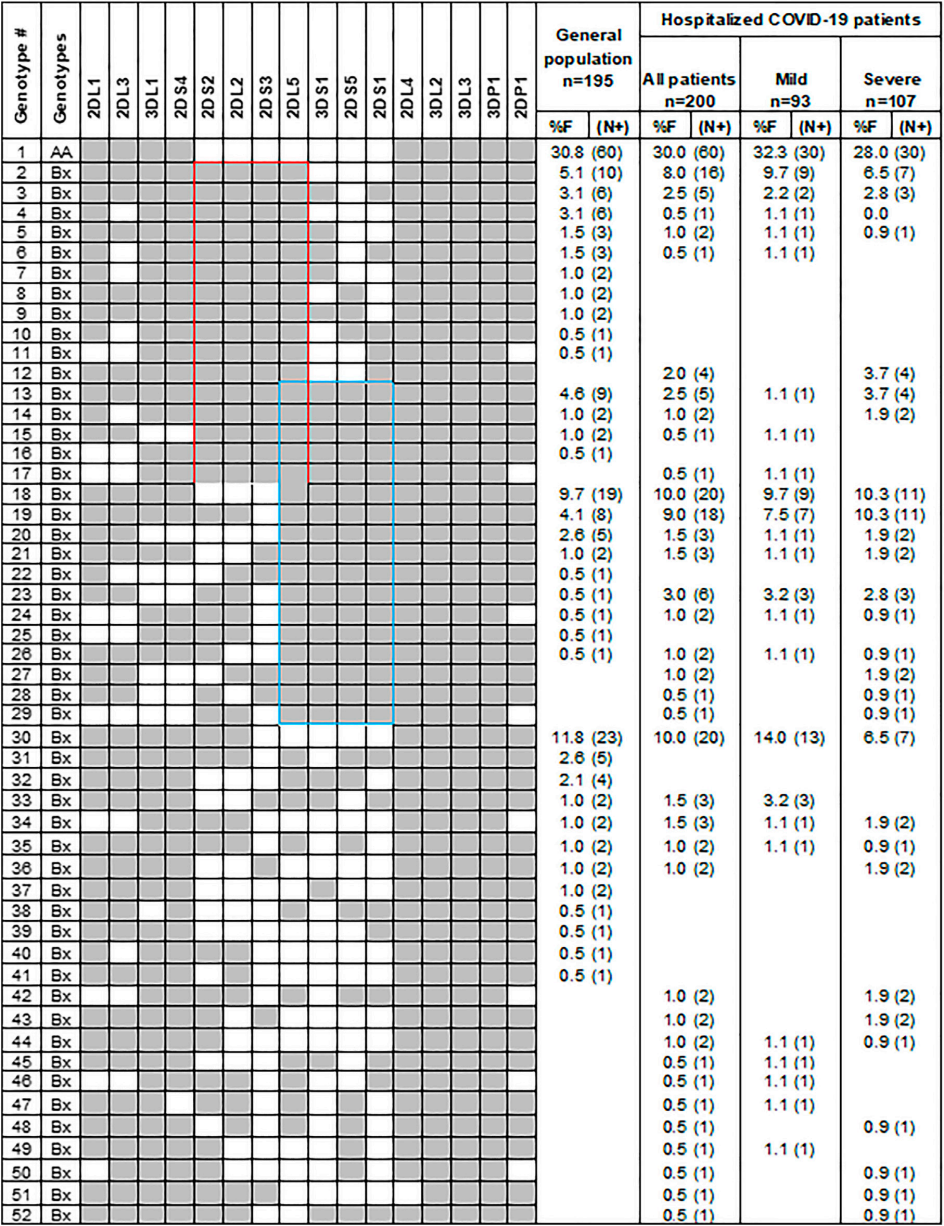
Gene content KIR genotypes in COVID-19 patients and controls. Fifty-two distinct KIR genotypes differ from each other by the presence (shaded box) or absence (white box) of 16 KIR genes. The frequency (%F) of each genotype is expressed as a percentage and defined as the number of individuals having that specific genotype (N) divided by the number of individuals studied (n) in each group. Red and blue boxes, respectively, mark the C4 and T4 linkage groups.

To investigate the role of variable KIR genes and HLA class I ligands in conferring susceptibility to COVID-19, we compared the frequency of KIR genes, KIR genotypes, and HLA class I ligands between patients and the general population. The frequency of individual KIR genes and genotypes are comparable between patients and the general population (Figure 2; Table 2). Among the HLA class I ligands, only HLA-A3 was significantly decreased in patients [11.5 vs. 26.2%, *p* = 0.0003, Odds ratio (OR) = 0.37, 95% confidence interval (CI) = 0.21–0.63].

**TABLE 2:** Frequency of KIR genotypes, KIR genes, and HLA class I ligands in the control group and COVID-19 patients.

	Hospitalized COVID-19 patients		
	All patients (A) *n* = 200	Mild (M) *n* = 93	Severe (S) n = 107	Controls (C) *n* = 195	Comparisons: *p*-value, OR (95% CI)
*KIR* Genotypes	**%F(N+)**	**%F (N+)**	**%F (N+)**	**%F (N+)**	
AA genotypes	30.0 (60)	32.3 (30)	28.0 (30)	30.8 (60)	
Bx genotypes	70.0 (140)	67.7 (63)	72.0 (77)	69.2 (140)	
C4 cluster	19.0 (38)	18.3 (17)	19.6 (21)	25.7 (50)	
T4 cluster	33.5 (67)	28.0 (26)	**38.3 (41)**	**27.2 (53)**	**SxC:** 0.0001, 4.3 (2.6–7.1)
*B haplotype-associated genes*					
*Centromeric genes*					
*2DS2*	52.5 (105)	51.6 (48)	53.3 (57)	49.7 (97)	
*2DL2*	49.5 (99)	48.4 (45)	50.5 (54)	49.2 (96)	
*2DS3*	26.0 (52)	22.6 (21)	29.0 (31)	28.2 (55)	
*2DL5*	53.5 (107)	50.5 (47)	56.1 (60)	52.8 (103)	
*Telomeric genes*					
*3DS1*	40.0 (80)	36.6 (34)	43.0 (46)	39.5 (77)	
*2DS5*	38.0 (76)	31.2 (29)	43.9 (47)	35.9 (70)	
*2DS1*	42.5 (85)	36.6 (34)	47.7 (51)	37.4 (73)	
*3DS1* ^*+*^ *2DS5* ^*+*^	34.0 (68)	28.0 (26)	39.3 (42)	30.3 (59)	
*3DS1* ^*+*^ *2DS1* ^*+*^	39.0 (78)	35.5 (33)	42.1 (45)	32.3 (63)	
*2DS1* ^*+*^ *2DS5* ^*+*^	35.0 (70)	**28.0 (26)**	**41.1 (44)**	30.8 (60)	**SxM:** 0.05, 1.8 (1–3.3)
*3DS1* ^*+*^ *2DS5* ^*+*^ *2DS1* ^*+*^	33.5 (67)	28.0 (26)	**38.3 (41)**	**27.2 (53)**	**SxC:** 0.046, 1.7 (1.0–2.7)
*A haplotype-associated genes*					
*2DL1*	94.5 (189)	95.7 (89)	93.5 (100)	96.9 (189)	
*2DL3*	93.0 (186)	93.5 (87)	92.5 (99)	88.7 (173)	
*3DL1*	93.0 (186)	94.6 (88)	91.6 (98)	94.9 (185)	
*2DS4*	92.0 (184)	93.5 (87)	90.7 (97)	94.9 (185)	
*Pseudogenes and Framework genes*					
*2DP1*	94.5 (189)	95.7 (89)	93.5 (100)	98.0 (191)	
*3DP1*	100.0 (200)	100.0 (93)	100.0 (107)	100.0 (195)	
*2DL4*	99.5 (199)	100.0 (93)	99.1 (106)	100.0 (195)	
*3DL2*	100.0 (200)	100.0 (93)	100.0 (107)	100.0 (195)	
*3DL3*	100.0 (200)	100.0 (93)	100.0 (107)	100.0 (195)	
*KIR-binding HLA class I ligands*					
C1	86.0 (172)	86.0 (80)	86.0 (92)	80.0 (156)	
C2	58.5 (117)	64.5 (60)	53.3 (57)	58.5 (114)	
Bw4	48.0 (96)	46.2 (43)	49.5 (53)	55.9 (109)	
Bw4 I80	34.5 (69)	34.4 (32)	34.6 (37)	34.4 (67)	
Bw4 T80	22.0 (44)	**17.2 (16)**	26.2 (28)	**30.3 (59)**	**MxC:** 0.019, 0.48 (0.3–0.89)
A3/11	**25.0 (50)**	26.9 (25)	**23.4 (25)**	**35.4 (69)**	**AxC:** 0.025, 0.61 (0.39–0.94); **SxC:** 0.032, 0.56 (0.33–0.95)
A3	**11.5 (23)**	14.0 (13)	9.3 (10)	**26.2 (51)**	**AxC:** 0.0003, 0.37 (0.21–0.63)
A11	15.0 (30)	12.9 (12)	16.8 (18)	11.3 (22)	

Frequency (%F) of each genotype is expressed as a percentage and defined as the number of individuals having the genotype (N+) divided by the number of individuals studied (n) in the study group;OR, Odds ratio; CI, Confidence interval; Comparisons: AxC, all patients vs. controls, MxC, mild vs. controls, SxC, severe vs. controls, SxM, sever vs. mild. Values shows significant difference are shown in bold.

To investigate the receptor-ligand combinatorial effect in predisposing COVID-19, we compared the frequency of individuals carrying specific KIR genes and their cognate HLA class I ligands between the COVID-19 patients and the general population. The frequency of both three Ig-domain containing inhibitory KIRs and their cognate HLA class I ligand combinations, such as KIR3DL1^+^HLA-Bw4^+^ (44.5 vs. 55.4%, *p* = 0.03, OR = 0.65, CI = 0.43–0.96) and KIR3DL2^+^HLA-A3/11^+^ (25 vs. 35.4%, *p* = 0.02, OR = 0.6, CI = 0.4–0.94), were significantly decreased in patients hospitalized with COVID-19 compared to the general population controls (Table 3). More patients with COVID-19 lack at least one of these KIR^+^HLA^+^ combinations compared to the general population controls (60 vs. 75.4%, *p* = 0.001, OR = 0.49, CI = 0.32–0.75). Notably, 40% of the COVID-19 patients were negative for both KIR3DL1^+^HLA-Bw4^+^ and KIR3DL2^+^HLA-A3/11^+^ combinations compared to 24.6% of the general population (OR = 2.04, *p* = 0.001, CI = 1.33–3.14).

**TABLE 3:** Frequency of KIR and HLA class I ligand combination in the control group and COVID-19 patients.

	Hospitalized COVID-19 patients		
	All patients (A) *n* = 200	Mild (M) *n* = 93	Severe (S) *n* = 107	Controls (C) *n* = 195	Comparisons: p-value, OR (95% CI)
Inhibitory KIR + HLA class I ligand	**%F (N+)**	**%F (N+)**	**%F (N+)**	**%F (N+)**	
2DL1^+^C2^+^	55.5 (111)	62.4 (58)	49.5 (53)	55.4 (108)	
2DL2^+^C1^+^	42.5 (85)	43.0 (40)	42.1 (45)	37.9 (74)	
2DL3^+^C1^+^	80.0 (160)	79.6 (74)	80.4 (86)	72.3 (141)	
3DL1^+^Bw4^+^	**44.5 (89)**	45.2 (42)	43.9 (47)	**55.4 (108)**	**AxC:** 0.03, 0.65 (0.43–0.96)
3DL1^+^Bw4 I80^+^	32.5 (65)	34.4 (32)	30.8 (33)	32.8 (64)	
3DL1^+^Bw4 T80^+^	**18.5 (37)**	**15.1 (14)**	21.5 (23)	**27.7 (54)**	**AxC:** 0.03, 0.6 (0.37–1.0); **MxC:** 0.02, 0.46 (0.24–0.9)
3DL2^+^A3/A11^+^	**25.0 (50)**	26.9 (25)	**23.4 (25)**	**35.4 (69)**	**AxC:** 0.02, 0.6 (0.4–0.94); **SxC:** 0.03, 0.56 (0.3–0.95)
3DL2^+^A3^+^	**11.5 (23)**	**15.1 (14)**	**8.4 (9)**	**26.1 (51)**	**AxC:** 0.0003, 0.37 (0.21–0.63); **MxC:** 0.037, 0.5 (0.26–0.96); **SxC:** 0.0004, 0.26 (0.1–0.6)
3DL2^+^A11^+^	15.0 (30)	12.9 (12)	16.8 (18)	11.3 (22)	
3DL1^+^Bw4^+^ and 3DL2^+^A3/A11^+^	9.5 (19)	10.8 (10)	8.4 (9)	15.4 (30)	
3DL1^+^Bw4^+^ and/or 3DL2^+^A3/A11^+^	**60.0 (120)**	**61.3 (57)**	**58.9 (63)**	**75.4 (147)**	**AxC:** 0.001, 0.49 (0.32–0.75); **MxC:** 0.015, 0.52 (0.3–0.9); **SxC:** 0.003, 0.5 (0.28–0.77)
(3DL1^+^ Bw4^+^)^-^ or (3DL2^+^A3/A11^+^)^-^	50.5 (101)	50.5 (47)	50.5 (54)	60.0 (117)	
(3DL1^+^Bw4^+^)^-^ and (3DL2^+^A3/A11^+^)^-^	**40.0 (80)**	**38.7 (36)**	**41.1 (44)**	**24.6 (48)**	**AxC:** 0.001, 2.04 (1.33–3.14); **MxC:** 0.015, 1.93 (1.1–3.3); **SxC:** 0.003, 2.14 (1.3–3.5)
Activating KIR + HLA class I ligand					
3DS1^+^Bw4^+^	19.5 (39)	18.3 (17)	20.6 (22)	21.5 (42)	
2DS1^+^C2^+^	25.5 (51)	24.7 (23)	26.2 (28)	22.5 (44)	
2DS2^+^C1^+^	45.0 (90)	45.2 (42)	44.9 (48)	37.9 (74)	
2DS2^+^A11^+^	7.5 (15)	5.4 (5)	9.3 (10)	4.6 (9)	
2DS5^+^C2^+^	23.5 (47)	21.5 (20)	25.2 (27)	22.1 (43)	
Inhibitory KIR + HLA class I ligand + Activating KIR					
3DL1^+^Bw4^+^ and 3DS1^-^	28.5 (57)	28.0 (26)	29.0 (31)	36.9 (72)	
3DL1^+^Bw4^+^ and 3DS1^+^	16.0 (32)	17.2 (16)	15.0 (16)	18.5 (36)	
2DL1^+^C2^+^ and 2DS1^-^	32.0 (64)	**39.8 (37)**	**25.2 (27)**	34.9 (68)	**SxM:** 0.03, 0.51 (0.23–0.93)
2DL1^+^C2^+^ and 2DS1^+^	23.5 (47)	22.6 (21)	24.3 (26)	21.0 (41)	
2DL1^+^C2^+^ and 2DS5^-^	34.0 (68)	**41.9 (39)**	**27.1 (29)**	34.9 (68)	**SxM:** 0.03, 0.51 (0.28–0.93)
2DL1^+^C2^+^ and 2DS5^+^	21.5 (43)	20.4 (19)	22.4 (24)	20.5 (40)	
2DL1^+^C2^+^ and 2DS1^-^2DS5^-^	29.5 (59)	**36.6 (34)**	**23.4 (25)**	31.3 (61)	**SxM:** 0.04, 0.53 (0.29–0.98)
2DL1^+^C2^+^ and 2DS1^+^2DS5^+^	19.0 (38)	17.2 (16)	20.6 (22)	17.4 (34)	

Frequency (%F) of each genotype is expressed as a percentage and defined as the number of individuals having the genotype (N+) divided by the number of individuals studied (n) in the study group;OR, Odds ratio; CI, Confidence interval; Comparisons: AxC, all patients vs. controls, MxC, mild vs. controls, SxC, severe vs. controls, SxM, sever vs. mild. Values shows significant difference are shown in bold.

We then examined the subsets of HLA-Bw4 and HLA-A3/11 ligands with their respective KIRs (Table 3). A significantly lower incidence of the low-affinity KIR3DL1^+^HLA-Bw4 T80^+^ combination (18.5 vs. 27.7%, *p* = 0.03, OR = 0.6, CI = 0.37–1.0) was observed in COVID-19 patients than the general population. Similarly, the frequency of the KIR3DL2^+^HLA-A3^+^ combination (11.5 vs. 26.1%, *p* = 0003, OR = 0.37, CI = 0.21–0.63) was significantly lower in COVID-19 patients than in the general population controls. The frequencies of KIR3DL1^+^HLA-Bw4 I80^+^ and KIR3DL2^+^HLA-A11^+^ combinations were comparable between patients and the general population controls. The allelic sequence variant compositions of HLA-A3 and Bw4 T80 + groups are comparable to those of the general population (Cao et al., 2001; Hurley et al., 2020).

### Activating KIRs 2DS1^+^2DS5^+^ are Associated With the Risk of Developing Severe COVID-19

To investigate the role of variable KIR and HLA class I ligands in modulating the severity of COVID-19 disease, we compared the frequency of KIR genes, HLA class I ligands, and KIR-HLA combinations between patients with mild or severe COVID-19 illness. Although statistically not significant, the incidence of three activating KIR genes (i.e., 3DS1, 2DS1, 2DS5) located at the telomeric half of KIR B-haplotypes was increased in patients developing severe COVID-19 disease compared to those developing mild disease (Table 2). Notably, the co-occurrence of KIR2DS1^+^KIR2DS5^+^ genes was significantly increased in patients developing severe COVID-19 illness than those developing mild disease (41.1 vs. 28%, *p* = 0.05, OR = 1.8, CI = 1.0–3.3). Moreover, those with KIR2DS1^+^2DS5^+^ but lacking either KIR3DL1^+^HLA-Bw4^+^ combination (27.1 vs. 14%, *p* = 0.025, OR = 2.3, CI = 1.1–4.7) or KIR3DL2^+^HLA-A3/11^+^ (30.8 vs. 22.6%, *p*=NS) combination or both KIR3DL1^+^HLA-Bw4^+^ and KIR2DL2^+^HLA-A3/11^+^ (40.2 vs. 25.8%, *p* = 0.03, OR = 1.9, CI = 1.05–3.53) combinations were more frequent in patients with severe disease compared to patients with mild disease (Table 4).

**TABLE 4:** Frequency of inhibitory KIR, cognate HLA class I ligands, and activating KIR gene combinations in the control group and COVID-19 patients.

Inhibitory KIR + HLA class I ligand + activating KIR	Hospitalized COVID-19 patients		
	All patients *n* = 200	Mild *n* = 93	Severe n = 107	Controls *n* = 195	Comparisons: p-value, OR (95% CI)
	**%F (N+)**	**%F (N+)**	**%F (N+)**	**%F (N+)**	
3DL1^+^Bw4^+^ and 2DS1^-^2DS5^-^	**30.5 (61)**	31.2 (29)	**29.9 (32)**	**41.5 (81)**	**AxC:** 0.02, 0.62 (0.41–0.94); **SxC:** 0.047, 0.6 (0.36–1)
(3DL1^+^Bw4^+^)^-^ and 2DS1^+^2DS5^+^	**21.0 (42)**	**14.0 (13)**	**27.1 (29)**	**13.3 (26)**	**AxC:** 0.04, 1.73 (1.0–2.9); **SxC:** 0.0036, 2.4 (1.3–4.4); **SxM:** 0.025, 2.3 (1.1–4.7)
3DL2^+^A3/11^+^ and 2DS1^-^2DS5^-^	17.0 (34)	21.5 (20)	13.1 (14)	22.1 (43)	
(3DL2^+^A3/11^+^)^-^ and 2DS1^+^2DS5^+^	**27.0 (54)**	22.6 (21)	**30.8 (33)**	**17.4 (34)**	**AxC:** 0.02, 1.75 (1.1–2.8); **SxC:** 0.008, 2.1 (1.2–3.7)
(3DL1^+^Bw4^+^)^-^ and/or (3DL2^+^A3/11^+^)^-^ 2DS1^+^2DS5^+^	**15.0 (30)**	10.8 (10)	**18.7 (20)**	**7.2 (14)**	**AxC:** 0.02, 2.28 (1.17–4.45); **SxC:** 0.003, 2.97 (1.4–6.2)
(3DL1^+^Bw4^+^)^-^ and (3DL2^+^A3/11^+^)^-^ and 2DS1^+^2DS5^+^	**33.5 (67)**	**25.8 (24)**	**40.2 (43)**	**23.6 (46)**	**AxC:** 0.03, 1.63 (1.04–2.53); **SxC:** 0.003, 2.2 (1.3–3.6); **SxM:** 0.03, 1.9 (1.05–3.53)
(3DL1^+^Bw4^+^)^-^ and/or (3DL2^+^A3/11^+^)^-^ and 2DL1^+^C2^+^ and 2DS1^+^2DS5^+^	**7.5 (15)**	6.5 (6)	**8.4 (9)**	**2.1 (4)**	**AxC:** 0.02, 3.87 (1.26–11.88); **SxC:** 0.016, 4.4 (1.3–14.6)
(3DL1^+^Bw4^+^)^-^ and/or (3DL2^+^A3/11^+^)^-^ and 2DL1^+^C2^+^ and 2DS1^-^2DS5^-^	8.5 (17)	11.8 (11)	5.6 (6)	8.2 (16)	
(3DL1^+^Bw4^+^)^-^ and (3DL2^+^A3/11^+^)^-^ and 2DL1^+^C2^+^ and 2DS1^+^2DS5^+^	**17.0 (34)**	15.1 (14)	**18.7 (20)**	**9.7 (19)**	**AxC:** 0.04, 1.90 (1.04–3.46); **SxC:** 0.03, 2.1 (1.1–4.2)
(3DL1^+^Bw4^+^)^-^ and (3DL2^+^A3/11^+^)^-^ and 2DL1^+^C2^+^ and 2DS1^-^2DS5^-^	26.5 (53)	31.2 (29)	22.4 (24)	26.7 (52)	

Frequency (%F) of each genotype is expressed as a percentage and defined as the number of individuals having the genotype (N+) divided by the number of individuals studied (n) in the study group;OR, Odds ratio; CI, Confidence interval; Comparisons: AxC, all patients vs. controls, MxC, mild vs. controls, SxC, severe vs. controls, SxM, sever vs. mild. Values shows significant difference are shown in bold.

### KIR2DL1^+^HLA-C2^+^ Combination in the Absence of the Activating Counterpart KIR2DS1 Protects From Developing Severe COVID-19 Illness

Among the five inhibitory KIR and cognate HLA class I combinations, only KIR2DL1^+^HLA-C2^+^ combinations occur more frequently in patients with mild disease than those with severe disease (62.4 vs. 49.5%); however, the difference was statistically insignificant (Table 3). We then examined the coexistence of inhibitory KIR, its cognate ligand, and the activating KIR counterpart known to bind the same HLA class I ligand. The KIR2DL1^+^HLA-C2^+^ combination without an activating counterpart of either KIR2DS1 (39.8 vs. 25.2%, *p* = 0.03, OR = 0.51, CI = 0.23–0.93) or KIR2DS5 (41.9 vs. 27.1%, *p* = 0.03, OR = 0.51, CI = 0.28–0.93) or both KIR2DS1 and KIR2DS5 were more frequent in mild COVID-19 cases than severe COVID-19 cases (36.6 vs. 23.4%, *p* = 0.04, OR = 0.53, CI = 0.29–0.98). The KIR3DL1^+^HLA-Bw4^+^ combination with and without the activating counterpart KIR3DS1 occurs in patients and the general population at a comparable frequency.

## Discussion

Despite the rapid spread of SARS-CoV-2 worldwide, a great majority of the infected individuals do not develop active disease, indicating the ability of human immune responses to contain the infection. The unanswered question is why only a fraction of infected individuals develop COVID-19 disease while most remain asymptomatic. NK cells are fast-acting effector lymphocytes that provide the crucial first line of defense against viral pathogens by their ability to kill infected cells and produce pro-inflammatory cytokines spontaneously (Biron et al., 1999). To understand the role of diverse KIR^+^HLA^+^ gene combinations in NK cell defense to SARS-CoV-2, we studied the host genetic polymorphism of KIR and their cognate HLA class I ligands in hospitalized COVID-19 patients and the general population. We observed that the KIR3DL1^+^HLA-Bw4^+^ and KIR3DL2^+^HLA-A3/11^+^ gene combinations were encountered at significantly lower frequency in COVID-19 patients than in the general population. Additionally, the activating KIR genes 2DS1 and 2DS5 were increased considerably in COVID-19 patients with severe illness compared to patients with mild disease.

**FIGURE 3:**
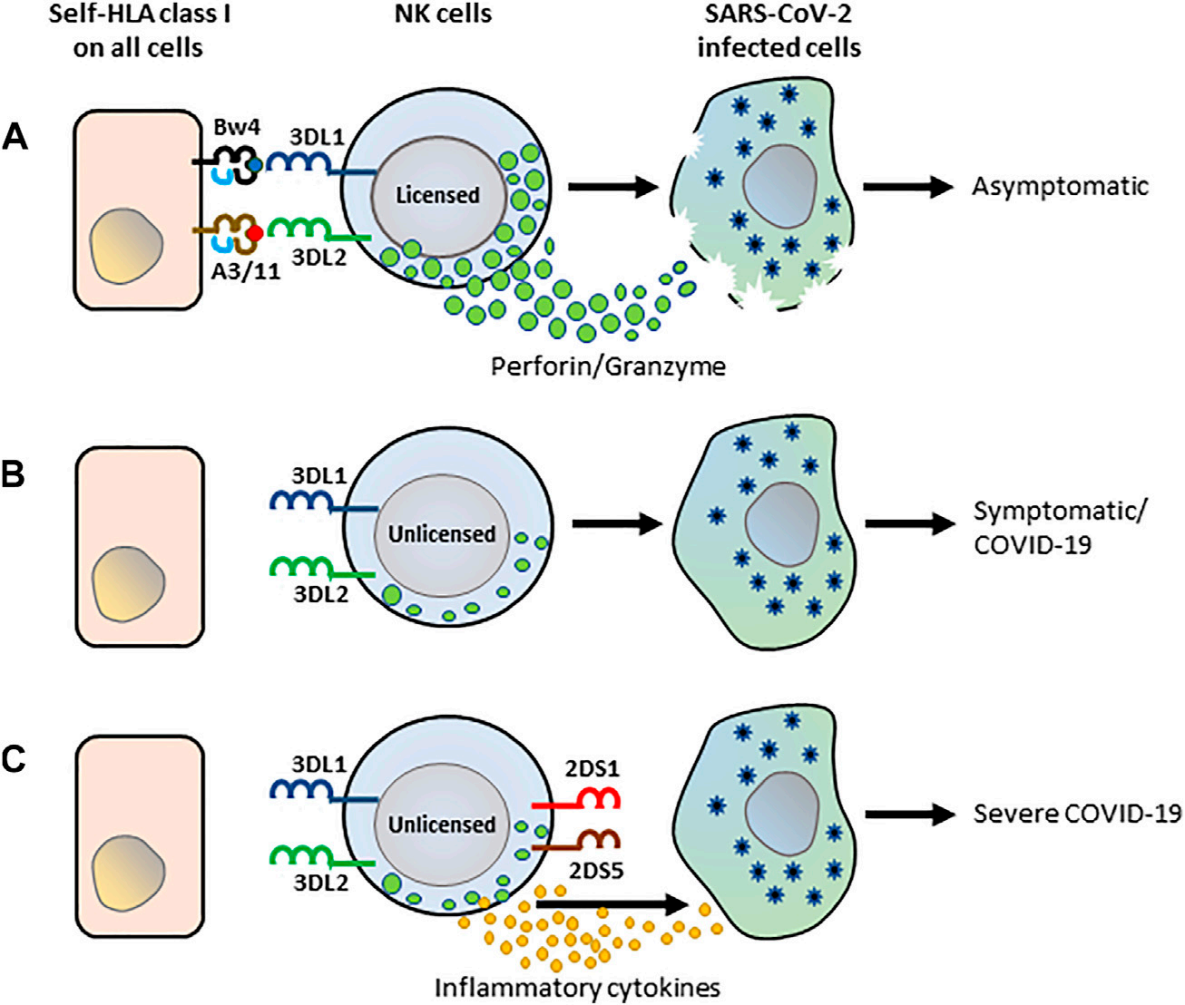
The schematic framework of KIR-HLA interactions by NK cells in anti-SARS-CoV-2 immunity. **(A)** Interactions of KIR3DL1 and KIR3DL2 with their cognate HLA class I ligands, HLA-Bw4 and HLA-A3/11 respectively, influence NK cell licensing and set a high threshold for NK cell lysis. The licensed NK cells recognize SARS-CoV-2-infected and trigger an antiviral response. **(B)** The unlicensed NK cells developed from those lacking KIR3DL1+Bw4+ and KIR3DL2+HLA-A3/11 + combinations are hyporesponsive and do not trigger an antiviral response, consequently leading to COVID-19 illness. **(C)** NK cells of patients lacking KIR3DL1+Bw4+ and KIR3DL2+HLA-A3/11 + combinations but expressing activating KIR2DS1 and 2DS5 presumably triggers abundant cytokines and chemokines arbitrating severe COVID-19.

The presence of both the inhibitory KIR receptors and their cognate HLA class I ligand is paradoxically associated with protection in several epidemiological studies (Rajagopalan and Long, 2005). Consistent with these findings, we observed a significantly decreased frequency of KIR3DL1^+^HLA-Bw4^+^ and KIR3DL2^+^HLA-A3/11^+^ gene combinations in hospitalized COVID-19 patients suggesting a protective role for these inhibitory KIR-HLA gene combinations to COVID-19. Presumably, the licensing mediated by these inhibitory KIR-HLA interactions is essential for producing potent NK cells to defend against SARS-CoV2 (Figure 3). Those missing these KIR-HLA gene combinations probably generate hyporesponsive NK cells and thus fail in mounting an effective anti-SARS-CoV2 defense. In support of this, blunted NK cell cytolytic activities were observed in severe COVID-19 patients (Osman et al., 2020; Bozzano et al., 2021). Moreover, COVID-19 patients were reported to have reduced NK cell functional markers such as CD107a+ (a degranulation marker), granzyme B, IFN-γ+, IL-2+, and TNF-α+ compared to healthy controls (Zheng et al., 2020). Future mechanistic studies are warranted to confirm the molecular basis of the decreased KIR3DL1^+^HLA-Bw4^+^ and KIR3DL2^+^HLA-A3/11^+^ gene combinations in dampening the effector functions of NK cells in COVID-19 patients.

Several functional studies support the role of KIR3DL1 and HLA-Bw4 interactions during the “licensing” process of NK cells. KIR3DL1^+^ NK cells isolated from HLA-Bw4^+^ individuals exhibited a higher amount of IFN-γ and cytotoxic potential than KIR3DL1^+^ NK cells isolated from HLA-Bw4^-^ individuals (Parsons et al., 2010). Similarly, another experiment demonstrated that KIR3DL1^+^ NK cells isolated from HLA-Bw4^+^ individuals showed higher degranulation potential against HLA-negative target cells compared to KIR3DL1^+^ NK cells isolated from HLA-Bw4^-^ individuals (Kim et al., 2008; Boudreau et al., 2013). These mechanistic studies strongly support the importance of KIR3DL1 recognizing self HLA-Bw4 during NK cell education to eliminate virally infected target cells. Future studies are needed to investigate the molecular mechanism underlying the KIR3DL1^+^HLA-Bw4^+^ interaction-driven licensed NK cell recognition of the SARS-CoV2-infected targets.

Consistent with our findings, the coinheritance of inhibitory KIR3DL1 and its cognate ligand HLA-Bw4 is associated with clearance of several viral infections (Bashirova et al., 2006), including conferring protection against influenza A (H1N1/09) virus infection (La et al., 2011). Furthermore, the combined genotype of KIR3DL1^+^ and HLA-Bw4^+^ was strongly associated with slower progression to acquired immunodeficiency syndrome (AIDS) in human immunodeficiency virus (HIV) infection (Martin and Carrington, 2013; Maruthamuthu et al., 2018). Protection from the progression of cervical neoplasia, a disease caused by the human papillomavirus, is correlated with the pairing of KIR3DL1 and its cognate ligand HLA-Bw4 (Carrington et al., 2005). Altogether, the KIR3DL1^+^HLA-Bw4^+^ combination protects an array of viral infections, including SARS-CoV2, H1N1/09, HIV, and human papillomavirus.

The functional interaction of KIR3DL2 with HLA-A3/11 is poorly studied, and this interaction is weaker than the other KIRs with HLA-C and HLA-B ligands (Dohring et al., 1996). Since KIR3DL2 is a framework gene present in all individuals, the functional role of the receptor is arguably dependent on its interaction with HLA-A3/11 (Sun et al., 2021). Although the mechanism of anti-SARS-CoV2 activity rendered by inhibitory KIR3DL2^+^HLA-A3/11^+^ interaction is unclear, our results indicate that NK cell recognition through 3DL2^+^HLA-A3^+^ interaction may trigger more robust NK cytolysis when HLA-A3 is presented with viral peptides (Hansasuta et al., 2004).

In our study, specific activating KIR genes located in the telomeric half of KIR B-haplotypes were found to be more frequent in patients with severe COVID-19 illness. Specifically, the coexistence of KIR2DS1 and KIR2DS5 genes were more frequent in severe COVID-19 patients. Although the ligand is unknown, the mechanistic studies using anti-KIR2DS5 antibodies revealed that the activation of KIR2DS5 triggers both NK cell cytotoxicity and IFN-γ release (Della Chiesa et al., 2008). Activating KIR2DS1 has been shown to bind HLA-C2, the same ligand as their structurally related inhibitory counterpart KIR2DL1, but at low affinity (Biassoni et al., 1997). Moreover, KIR2DS1 has displayed a certain degree of peptide selectivity in its binding to HLA-C2 (Stewart et al., 2005), and thus not always sufficient to trigger NK effector responses (Moretta et al., 1995). The activating KIR2DS1 and KIR2DS5 may recognize the SARS-CoV2 infected cells and contribute to abundant pro-inflammatory cytokines that facilitate the development of severe COVID-19 illness (Del Valle et al., 2020) (Figure 3).

An increased frequency of KIR2DL1^+^HLA-C2^+^ combination, particularly without the activating counterpart KIR2DS1 or KIR2DS5 in mild COVID-19 cases than severe COVID-19 cases, suggests a protective role for KIR2DL1^+^HLA-C2^+^ against developing severe COVID-19. Perhaps the protection is conferred by NK cell licensing driven by KIR2DL1^+^HLA-C2^+^ interaction. In the presence of activating KIR2DS1 or KIR2DS5, the protective effect of KIR2DL1^+^HLA-C2^+^ engagement is lifted. This data is in line with our previous finding of increased prevalence of KIR2DL1^+^KIR2DS1^+^HLA-C2 gene constellations in patients with advanced breast cancer (Ashouri et al., 2021). In mouse models, prolonged NKG2D engagement impairs NK cell function (Coudert et al., 2008). When mouse NK cells are chronically exposed to the ligand of the activating receptor (Ly49H), these cells become unresponsive to infected target cells (Sun and Lanier, 2008). In acute myeloid leukemia, an anti-leukemic effect was shown in patients who received hematopoietic stem-cell transplantation from KIR2DS1^+^HLA-C1/C1^+^ or KIR2DS1^+^HLA-C1/C2^+^ donors, whereas grafts from donors who were homozygous for HLA-C2 did not provide any advantage (Venstrom et al., 2012). The interaction of KIR2DS1 with high levels of its ligands HLA-C2 reduces KIR2DS1^+^ NK cell functionality (Venstrom et al., 2012). Thus, the ligation of KIR2DS1 with HLA-C2 on infected cells may result in overactivated or anergic KIR2DS1^+^ NK cells that may have a deleterious effect on COVID-19 severity.

Only five studies have investigated the impact of KIR receptors on SARS-CoV-2 infection (Beksac et al., 2021; Bernal et al., 2021; Lesan et al., 2021; Littera et al., 2021; Hajeer et al., 2022). In contrast to our findings, a study with a small number of 16 patients from the German Caucasian cohort revealed that patients with activating KIR2DS5 were recovered from COVID-19 in a shorter time than the individuals negative for the KIR2DS5 gene (Lesan et al., 2021). Although the ligand for activating KIR2DS2 is unknown, the combination of KIR2DS2 and HLA-C1 was found to have a potent protective effect against adverse outcomes of COVID-19 in Sardinia, a large Italian island in the Mediterranean Sea (Littera et al., 2021). Moreover, this study also found a significant association between AA KIR-haplotypes and patients with severe COVID-19 disease compared to symptomatic-paucisymptomatic patients. In contrast to the Sardinian study, Bx genotypes were associated with COVID-19 patients in Soudhi Arabian population (Hajeer et al., 2022). A study in Turkey revealed an association between COVID-19 and specific centromeric and telomeric halves of KIR haplotypes with HLA class I ligands (Beksac et al., 2021). However, this study did not determine the frequencies of specific KIR and cognate HLA class I ligand combinations. Higher frequencies for KIR2DS4 in severe COVID-19 patients and KIR3DS1^+^HLA-B*15:01^+^ in mild/moderate cases of COVID-19 than in control in Spanish indicate a potentially detrimental effect of activating KIR in COVID-19 (Bernal et al., 2021). However, none of these genes and combinations were found to be significant in our study, probably because of the limited sample sizes in these other studies. In addition to small sample sizes, the KIR and HLA gene frequency among different human populations varies largely (Du et al., 2007; Rajalingam et al., 2008; Ashouri et al., 2009), explaining the observed discrepancies. Notably, none of these studies analyzed KIR3DL2^+^HLA-A3/11^+^ combinations.

A few clinical trials are ongoing with a straightforward assumption that infusing *ex vivo* expanded NK cells into COVID-19 patients can reinstate immune capacity and increase chances of recovery (ClinicalTrials.gov Identifier: NCT04344548, NCT04365101, NCT04280224). Replenishing expanded NK cells is risky because the supplementary NK cells may become detrimental by producing a massive amount of cytokines and chemokines instead of playing a beneficial antiviral killer response. Considering the novel finding from the current study, we propose to use NK cells from the donor carrying KIR3DL1^+^HLA-Bw4^+^ and KIR3DL2^+^HLA-A3/11^+^ interactions but lacking activating KIR receptors 2DS1 and 2DS5.

A limitation of the present study was the utilization of samples from the pre-pandemic general population as a control. Studying the KIR-HLA combination frequency in asymptomatic SARS-CoV2 positive individuals would be a more appropriate control group. However, due to testing strategy limitations, identifying these subjects was difficult during the early stages of the pandemic. All five previous studies investigated the role of KIR receptors in COVID-19 also used the general population controls collected pre-pandemic, signifying challenges associated with these initial correlative studies. In theory, all control subjects included in this study should have been infected with SARS-CoV-2. Based on the epidemiological data (Menachemi et al., 2021), assuming all controls were infected, only 2.1% of the controls (n = 4) are estimated to have been hospitalized for COVID-19 care, and the remaining 97.9% (n = 191) remain to be asymptomatic. Therefore, comparing this pre-pandemic general population with 200 patients hospitalized for COVID-19 care is a seemingly reasonable approach. Our study cohorts are insufficient to evaluate the impact of multiple KIR-HLA combinations in different clinical phenotypes. Future systematic studies using multivariate analysis on larger cohorts are required to substantiate our findings and determine the role of multiple KIR-HLA pairs on the diverse outcomes of SARS-CoV2 infection. Nonetheless, our data support our hypothesis and provide novel insights into the molecular mechanisms for dual functions of NK cells in responding to acute SARS-CoV2 infections and contributing to COVID-19 immunopathology. Presumably, the reduced antiviral defense due to the absence of KIR3DL1^+^HLA-Bw4^+^ and KIR3DL2^+^HLA-A3/11^+^ interactions coupled with exuberant hyperinflammatory response mediated by activating KIR2DS1 and 2DS5 arbitrate the development of severe COVID-19. Future functional studies are warranted to elucidate the mechanism of these host genetic associations of COVID-19.

## Data Availability

The original contributions presented in the study are included in the article, further inquiries can be directed to the corresponding author.

## References

[B1] Anfossi N. André P. Guia S. Falk C. S. Roetynck S. Stewart C. A. (2006). Human NK Cell Education by Inhibitory Receptors for MHC Class I. Immunity 25 (2), 331–342. 10.1016/j.immuni.2006.06.013 16901727

[B2] Ashouri E. Farjadian S. Reed E. F. Ghaderi A. Rajalingam R. (2009). KIR Gene Content Diversity in Four Iranian Populations. Immunogenetics 61 (7), 483–492. 10.1007/s00251-009-0378-7 19521696 PMC2706385

[B3] Ashouri E. Rajalingam K. Barani S. Farjadian S. Ghaderi A. Rajalingam R. (2021). Coexistence of Inhibitory and Activating Killer-Cell Immunoglobulin-like Receptors to the Same Cognate HLA-C2 and Bw4 Ligands Confer Breast Cancer Risk. Sci. Rep. 11 (1), 7932. 10.1038/s41598-021-86964-y 33846431 PMC8041876

[B4] Bao C. Tao X. Cui W. Hao Y. Zheng S. Yi B. (2021). Natural Killer Cells Associated with SARS-CoV-2 Viral RNA Shedding, Antibody Response and Mortality in COVID-19 Patients. Exp. Hematol. Oncol. 10 (1), 5. 10.1186/s40164-021-00199-1 33504359 PMC7839286

[B5] Bashirova A. A. Martin M. P. McVicar D. W. Carrington M. (2006). The Killer Immunoglobulin-like Receptor Gene Cluster: Tuning the Genome for Defense. Annu. Rev. Genom. Hum. Genet. 7, 277–300. 10.1146/annurev.genom.7.080505.115726 16824023

[B6] Beksac M. Akin H. Y. Gencer-Oncul E. B. Yousefzadeh M. Cengiz Seval G. Gulten E. (2021). A Model Integrating Killer Immunoglobulin-like Receptor (KIR) Haplotypes for Risk Prediction of COVID-19 Clinical Disease Severity. Immunogenetics 73 (6), 449–458. 10.1007/s00251-021-01227-4 34536086 PMC8449213

[B7] Bernal E. Gimeno L. Alcaraz M. J. Quadeer A. A. Moreno M. Martínez-Sánchez M. V. (2021). Activating Killer-Cell Immunoglobulin-like Receptors Are Associated with the Severity of Coronavirus Disease 2019. J. Infect. Dis. 224 (2), 229–240. 10.1093/infdis/jiab228 33928374 PMC8135764

[B8] Bhala N. Curry G. Martineau A. R. Agyemang C. Bhopal R. (2020). Sharpening the Global Focus on Ethnicity and Race in the Time of COVID-19. Lancet. 10.1016/S0140-6736(20)31102-8 PMC7211499 32401716

[B9] Biassoni R. Pessino A. Malaspina A. Cantoni C. Bottino C. Sivori S. (1997). Role of Amino Acid Position 70 in the Binding Affinity of p50.1 and p58.1 Receptors for HLA-Cw4 Molecules. Eur. J. Immunol. 27 (12), 3095–3099. 10.1002/eji.1830271203 9464792

[B10] Biron C. A. Byron K. S. Sullivan J. L. (1989). Severe Herpesvirus Infections in an Adolescent without Natural Killer Cells. N. Engl. J. Med. 320 (26), 1731–1735. 10.1056/NEJM198906293202605 2543925

[B11] Biron C. A. Nguyen K. B. Pien G. C. Cousens L. P. Salazar-Mather T. P. (1999). Natural Killer Cells in Antiviral Defense: Function and Regulation by Innate Cytokines. Annu. Rev. Immunol. 17, 189–220. 10.1146/annurev.immunol.17.1.189 10358757

[B12] Björkström N. K. Strunz B. Ljunggren H.-G. (2021). Natural Killer Cells in Antiviral Immunity. Nat. Rev. Immunol. 10.1038/s41577-021-00558-3 PMC8194386 34117484

[B13] Blokhuis J. H. Hilton H. G. Guethlein L. A. Norman P. J. Nemat-Gorgani N. Nakimuli A. (2017). KIR2DS5 Allotypes that Recognize the C2 Epitope of HLA-C Are Common Among Africans and Absent from Europeans. Immun. Inflamm. Dis. 5 (4), 461–468. 10.1002/iid3.178 28685972 PMC5691316

[B14] Boudreau J. E. Le Luduec J.-B. Hsu K. C. (2013). KIR3DL1 and HLA-Bw4 Allotypes Predict the Extent of NK Cell Licensing. Blood 122 (21), 1043. 10.1182/blood.V122.21.1043.1043

[B15] Bozzano F. Dentone C. Perrone C. Di Biagio A. Fenoglio D. Parodi A. (2021). Extensive Activation, Tissue Trafficking, Turnover and Functional Impairment of NK Cells in COVID-19 Patients at Disease Onset Associates with Subsequent Disease Severity. Plos Pathog. 17 (4), e1009448. ARTN e1009448. 10.1371/journal.ppat.1009448 33861802 PMC8081333

[B16] Cao K. Hollenbach J. Shi X. Shi W. Chopek M. Fernández-Viña M. A. (2001). Analysis of the Frequencies of HLA-A, B, and C Alleles and Haplotypes in the Five Major Ethnic Groups of the United States Reveals High Levels of Diversity in These Loci and Contrasting Distribution Patterns in These Populations. Hum. Immunol. 62 (9), 1009–1030. 10.1016/s0198-8859(01)00298-1 11543903

[B17] Carlomagno S. Falco M. Bono M. Alicata C. Garbarino L. Mazzocco M. (2017). KIR3DS1-Mediated Recognition of HLA-*B51: Modulation of KIR3DS1 Responsiveness by Self HLA-B Allotypes and Effect on NK Cell Licensing. Front. Immunol. 8, 581. 10.3389/fimmu.2017.00581 28603523 PMC5445109

[B18] Carrington M. Wang S. Martin M. P. Gao X. Schiffman M. Cheng J. (2005). Hierarchy of Resistance to Cervical Neoplasia Mediated by Combinations of Killer Immunoglobulin-like Receptor and Human Leukocyte Antigen Loci. J. Exp. Med. 201 (7), 1069–1075. 10.1084/jem.20042158 15809352 PMC2213116

[B19] Chewning J. H. Gudme C. N. Hsu K. C. Selvakumar A. Dupont B. (2007). KIR2DS1-positive NK Cells Mediate Alloresponse against the C2 HLA-KIR Ligand Group *In Vitro* . J. Immunol. 179 (2), 854–868. 10.4049/jimmunol.179.2.854 17617576

[B20] Chiesa M. D. Romeo E. Falco M. Balsamo M. Augugliaro R. Moretta L. (2008). Evidence that the KIR2DS5 Gene Codes for a Surface Receptor Triggering Natural Killer Cell Function. Eur. J. Immunol. 38 (8), 2284–2289. 10.1002/eji.200838434 18624290

[B21] Coudert J. D. Scarpellino L. Gros F. Vivier E. Held W. (2008). Sustained NKG2D Engagement Induces Cross-Tolerance of Multiple Distinct NK Cell Activation Pathways. Blood 111 (7), 3571–3578. 10.1182/blood-2007-07-100057 18198346

[B22] Del Valle D. M. Kim-Schulze S. Huang H.-H. Beckmann N. D. Nirenberg S. Wang B. (2020). An Inflammatory Cytokine Signature Predicts COVID-19 Severity and Survival. Nat. Med. 26 (10), 1636–1643. 10.1038/s41591-020-1051-9 32839624 PMC7869028

[B23] Döhring C. Scheidegger D. Samaridis J. Cella M. Colonna M. (1996). A Human Killer Inhibitory Receptor Specific for HLA-A1,2. J. Immunol. 156 (9), 3098–3101. 8617928

[B24] Drake T. M. Riad A. M. Fairfield C. J. Egan C. Knight S. R. Pius R. (2021). Characterisation of In-Hospital Complications Associated with Covid-19 Using the Isaric Who Clinical Characterisation Protocol UK: a Prospective, Multicentre Cohort Study. Lancet 398 (10296), 223–237. 10.1016/S0140-6736(21)00799-6 34274064 PMC8285118

[B25] Du Z. Gjertson D. W. Reed E. F. Rajalingam R. (2007). Receptor-ligand Analyses Define Minimal Killer Cell Ig-like Receptor (KIR) in Humans. Immunogenetics 59 (1), 1–15. 10.1007/s00251-006-0168-4 17103212

[B27] Foley B. De Santis D. Lathbury L. Christiansen F. Witt C. (2008). KIR2DS1-mediated Activation Overrides NKG2A-Mediated Inhibition in HLA-C C2-Negative Individuals. Int. Immunol. 20 (4), 555–563. 10.1093/intimm/dxn013 18308713

[B28] Gumperz J. E. Litwin V. Phillips J. H. Lanier L. L. Parham P. (1995). The Bw4 Public Epitope of HLA-B Molecules Confers Reactivity with Natural Killer Cell Clones that Express NKB1, a Putative HLA Receptor. J. Exp. Med. 181 (3), 1133–1144. 10.1084/jem.181.3.1133 7532677 PMC2191933

[B29] Hajeer A. Jawdat D. Massadeh S. Aljawini N. Abedalthagafi M. S. Arabi Y. M. (2022). Association of KIR Gene Polymorphisms with COVID-19 Disease. Clin. Immunol. 234, 108911. 10.1016/j.clim.2021.108911 34929414 PMC8683215

[B30] Hansasuta P. Dong T. Thananchai H. Weekes M. Willberg C. Aldemir H. (2004). Recognition of HLA-A3 and HLA-A11 by KIR3DL2 Is Peptide-specific. Eur. J. Immunol. 34 (6), 1673–1679. 10.1002/eji.200425089 15162437

[B31] Hayley M. Bourbigot S. Booth V. (2011). Self-association of an Activating Natural Killer Cell Receptor, KIR2DS1. PLoS ONE 6 (8), e23052. 10.1371/journal.pone.0023052 21912587 PMC3166062

[B32] Hilton H. G. Vago L. Older Aguilar A. M. Moesta A. K. Graef T. Abi-Rached L. (2012). Mutation at Positively Selected Positions in the Binding Site for HLA-C Shows that KIR2DL1 Is a More Refined but Less Adaptable NK Cell Receptor Than KIR2DL3. J.I. 189 (3), 1418–1430. 10.4049/jimmunol.1100431 PMC3439511 22772445

[B33] Hurley C. K. Kempenich J. Wadsworth K. Sauter J. Hofmann J. A. Schefzyk D. (2020). Common, Intermediate and Well‐documented HLA Alleles in World Populations: CIWD Version 3.0.0. HLA 95 (6), 516–531. 10.1111/tan.13811 31970929 PMC7317522

[B34] Khakoo S. I. Carrington M. (2006). KIR and Disease: a Model System or System of Models? Immunol. Rev. 214, 186–201. 10.1111/j.1600-065x.2006.00459.x 17100885

[B35] Kim S. Sunwoo J. B. Yang L. Choi T. Song Y.-J. French A. R. (2008). HLA Alleles Determine Differences in Human Natural Killer Cell Responsiveness and Potency. Proc. Natl. Acad. Sci. 105 (8), 3053–3058. 10.1073/pnas.0712229105 18287063 PMC2268583

[B36] Kong D. Lee N. Dela Cruz I. D. Dames C. Maruthamuthu S. Golden T. (2021). Concurrent Typing of over 4000 Samples by Long-Range PCR Amplicon-Based NGS and rSSO Revealed the Need to Verify NGS Typing for HLA Allelic Dropouts. Hum. Immunol. 82 (8), 581–587. 10.1016/j.humimm.2021.04.008 33980471

[B37] Kucuksezer U. C. Aktas Cetin E. Esen F. Tahrali I. Akdeniz N. Gelmez M. Y. (2021). The Role of Natural Killer Cells in Autoimmune Diseases. Front. Immunol. 12, 622306. 10.3389/fimmu.2021.622306 33717125 PMC7947192

[B38] La D. Czarnecki C. El-Gabalawy H. Kumar A. Meyers A. F. A. Bastien N. (2011). Enrichment of Variations in KIR3DL1/S1 and KIR2DL2/L3 Among H1N1/09 ICU Patients: an Exploratory Study. PLoS One 6 (12), e29200. 10.1371/journal.pone.0029200 22216211 PMC3247251

[B39] Lanier L. L. (1998). NK Cell Receptors. Annu. Rev. Immunol. 16, 359–393. 10.1146/annurev.immunol.16.1.359 9597134

[B40] Lesan V. Bewarder M. Metz C. Becker A. Mang S. Regitz E. (2021). Killer Immunoglobulin-like Receptor 2DS5 Is Associated with Recovery from Coronavirus Disease 2019. ICMx 9 (1), 45. 10.1186/s40635-021-00409-4 34476598 PMC8412971

[B41] Littera R. Chessa L. Deidda S. Angioni G. Campagna M. Lai S. (2021). Natural Killer-Cell Immunoglobulin-like Receptors Trigger Differences in Immune Response to SARS-CoV-2 Infection. PLoS One 16 (8), e0255608. 10.1371/journal.pone.0255608 34352002 PMC8341547

[B42] Liu J. Xiao Z. Ko H. L. Shen M. Ren E. C. (2014). Activating Killer Cell Immunoglobulin-like Receptor 2DS2 Binds to HLA-A*11. Proc. Natl. Acad. Sci. 111 (7), 2662–2667. 10.1073/pnas.1322052111 24550293 PMC3932910

[B43] Martin M. P. Carrington M. (2013). Immunogenetics of HIV Disease. Immunol. Rev. 254 (1), 245–264. 10.1111/imr.12071 23772624 PMC3703621

[B44] Maruthamuthu S. Rajalingam R. Pandian K. Madasamy S. Manoharan M. Pitchai L. (2018). Inhibitory Natural Killer Cell Receptor KIR3DL1 with its Ligand Bw4 Constraints HIV-1 Disease Among South Indians. Aids 32 (18), 2679–2688. 10.1097/Qad.0000000000002028 30289808

[B45] Maucourant C. Filipovic I. Ponzetta A. Aleman S. Cornillet M. Hertwig L. (2020). Natural Killer Cell Immunotypes Related to COVID-19 Disease Severity. Sci. Immunol. 5 (50). 10.1126/sciimmunol.abd6832 PMC7665314 32826343

[B46] Menachemi N. Dixon B. E. Wools-Kaloustian K. K. Yiannoutsos C. T. Halverson P. K. (2021). How Many SARS-CoV-2-Infected People Require Hospitalization? Using Random Sample Testing to Better Inform Preparedness Efforts. J. Public Health Manag. Pract. 27 (3), 246–250. 10.1097/PHH.0000000000001331 33729203

[B47] Moretta A. Sivori S. Vitale M. Pende D. Morelli L. Augugliaro R. (1995). Existence of Both Inhibitory (P58) and Activatory (P50) Receptors for HLA-C Molecules in Human Natural Killer Cells. J. Exp. Med. 182 (3), 875–884. 10.1084/jem.182.3.875 7650491 PMC2192157

[B48] O'Connor G. M. Vivian J. P. Gostick E. Pymm P. Lafont B. A. P. Price D. A. (2015). Peptide-Dependent Recognition of HLA-B*57:01 by KIR3DS1. J. Virol. 89 (10), 5213–5221. 10.1128/JVI.03586-14 25740999 PMC4442525

[B49] Osman M. Faridi R. M. Sligl W. Shabani-Rad M.-T. Dharmani-Khan P. Parker A. (2020). Impaired Natural Killer Cell Counts and Cytolytic Activity in Patients with Severe COVID-19. Blood Adv. 4 (20), 5035–5039. 10.1182/bloodadvances.2020002650 33075136 PMC7594380

[B50] Parham P. (2005). MHC Class I Molecules and KIRs in Human History, Health and Survival. Nat. Rev. Immunol. 5 (3), 201–214. 10.1038/nri1570 15719024

[B51] Parsons M. S. Zipperlen K. Gallant M. Grant M. (2010). Killer Cell Immunoglobulin-like Receptor 3DL1 Licenses CD16-Mediated Effector Functions of Natural Killer Cells. J. Leukoc. Biol. 88 (5), 905–912. 10.1189/jlb.1009687 20664023

[B52] Pavlovich S. S. Lovett S. P. Koroleva G. Guito J. C. Arnold C. E. Nagle E. R. (2018). The Egyptian Rousette Genome Reveals Unexpected Features of Bat Antiviral Immunity. Cell 173 (5), 1098–1110 e1018. 10.1016/j.cell.2018.03.070 29706541 PMC7112298

[B53] Rajagopalan S. Long E. O. (2005). Understanding How Combinations of HLA and KIR Genes Influence Disease. J. Exp. Med. 201 (7), 1025–1029. 10.1084/jem.20050499 15809348 PMC2213130

[B54] Rajalingam R. (2018). Diversity of Killer Cell Immunoglobulin-like Receptors and Disease. Clin. Lab. Med. 38 (4), 637–653. 10.1016/j.cll.2018.08.001 30420058

[B55] Rajalingam R. Du Z. Meenagh A. Luo L. Kavitha V. J. Pavithra-Arulvani R. (2008). Distinct Diversity of KIR Genes in Three Southern Indian Populations: Comparison with World Populations Revealed a Link between KIR Gene Content and Pre-historic Human Migrations. Immunogenetics 60 (5), 207–217. 10.1007/s00251-008-0286-2 18369612

[B56] Saulquin X. Gastinel L. N. Vivier E. (2003). Crystal Structure of the Human Natural Killer Cell Activating Receptor KIR2DS2 (CD158j). J. Exp. Med. 197 (7), 933–938. 10.1084/jem.20021624 12668644 PMC2193886

[B26] Severe Covid-19 GWAS Group. Ellinghaus D. Degenhardt F. Bujanda L. Buti M. Albillos A. Invernizzi P. (2020). Genomewide Association Study of Severe Covid-19 with Respiratory Failure. N. Engl. J. Med. 383 (16), 1522–1534. 10.1056/NEJMoa2020283 32558485 PMC7315890

[B57] Sivori S. Carlomagno S. Falco M. Romeo E. Moretta L. Moretta A. (2011). Natural Killer Cells Expressing the KIR2DS1-Activating Receptor Efficiently Kill T-Cell Blasts and Dendritic Cells: Implications in Haploidentical HSCT. Blood 117 (16), 4284–4292. blood-2010-10-316125 [pii]. 10.1182/blood-2010-10-316125 21355085

[B58] Song J.-W. Zhang C. Fan X. Meng F.-P. Xu Z. Xia P. (2020). Immunological and Inflammatory Profiles in Mild and Severe Cases of COVID-19. Nat. Commun. 11 (1), 3410. 10.1038/s41467-020-17240-2 32641700 PMC7343781

[B59] Stetson D. B. Mohrs M. Reinhardt R. L. Baron J. L. Wang Z.-E. Gapin L. (2003). Constitutive Cytokine mRNAs Mark Natural Killer (NK) and NK T Cells Poised for Rapid Effector Function. J. Exp. Med. 198 (7), 1069–1076. 10.1084/jem.20030630 14530376 PMC2194220

[B60] Stewart C. A. Laugier-Anfossi F. Vely F. Saulquin X. Riedmuller J. Tisserant A. (2005). Recognition of Peptide-MHC Class I Complexes by Activating Killer Immunoglobulin-like Receptors. Proc. Natl. Acad. Sci. 102 (37), 13224–13229. 10.1073/pnas.0503594102 16141329 PMC1201584

[B61] Sun H. Martin T. G. Marra J. Kong D. Keats J. Macé S. (2021). Individualized Genetic Makeup that Controls Natural Killer Cell Function Influences the Efficacy of Isatuximab Immunotherapy in Patients with Multiple Myeloma. J. Immunother. Cancer 9 (7), e002958. 10.1136/jitc-2021-002958 34272304 PMC8287616

[B62] Sun J. C. Lanier L. L. (2008). Tolerance of NK Cells Encountering Their Viral Ligand during Development. J. Exp. Med. 205 (8), 1819–1828. 10.1084/jem.20072448 18606858 PMC2525590

[B63] Venstrom J. M. Pittari G. Gooley T. A. Chewning J. H. Spellman S. Haagenson M. (2012). HLA-C-Dependent Prevention of Leukemia Relapse by Donor ActivatingKIR2DS1. N. Engl. J. Med. 367 (9), 805–816. 10.1056/NEJMoa1200503 22931314 PMC3767478

[B64] Wang F. Nie J. Wang H. Zhao Q. Xiong Y. Deng L. (2020). Characteristics of Peripheral Lymphocyte Subset Alteration in COVID-19 Pneumonia. J. Infect. Dis. 221 (11), 1762–1769. 10.1093/infdis/jiaa150 32227123 PMC7184346

[B65] Winter C. C. Gumperz J. E. Parham P. Long E. O. Wagtmann N. (1998). Direct Binding and Functional Transfer of NK Cell Inhibitory Receptors Reveal Novel Patterns of HLA-C Allotype Recognition. J. Immunol. 161 (2), 571–577. 9670929

[B66] Zheng M. Gao Y. Wang G. Song G. Liu S. Sun D. (2020). Functional Exhaustion of Antiviral Lymphocytes in COVID-19 Patients. Cell Mol Immunol 17 (5), 533–535. 10.1038/s41423-020-0402-2 32203188 PMC7091858

[B67] Zhou Z. Ren L. Zhang L. Zhong J. Xiao Y. Jia Z. (2020). Heightened Innate Immune Responses in the Respiratory Tract of COVID-19 Patients. Cell Host & Microbe 27 (6), 883–890 e882. 10.1016/j.chom.2020.04.017 32407669 PMC7196896

